# Perturbomics: CRISPR–Cas screening-based functional genomics approach for drug target discovery

**DOI:** 10.1038/s12276-025-01487-0

**Published:** 2025-07-01

**Authors:** Byung-Sun Park, Mieun Lee, Jaeyeol Kim, Tackhoon Kim

**Affiliations:** 1 Medicinal Materials Research Center, Korea Institute of Science and Technology, Seoul, Republic of Korea; 2 Department of Life Sciences, Korea University, Seoul, Republic of Korea; 3 Division of Bio-Medical Science and Technology, Korea National University of Science and Technology, Daejeon, Republic of Korea

**Keywords:** Mutagenesis, High-throughput screening

## Abstract

Despite more than two decades since the completion of the first draft of the Human Genome Project, a substantial proportion of human genes remain poorly characterized in terms of their functions. Functional genomics aims to elucidate the roles and interactions of genes and genetic elements, providing insights into their involvement in various biological processes. In this context, the perturbomics approach—a systematic analysis of phenotypic changes resulting from gene function modulation—offers valuable insights into the function of unannotated genes. With the advent of CRISPR–Cas-based genome and epigenome editing, CRISPR screens have become the method of choice for perturbomics studies, enabling the identification of target genes whose modulation may hold therapeutic potential for diseases such as cancer, cardiovascular disorders and neurodegeneration. These findings contribute to the development of targeted drug therapies and the design of gene and cell therapies for regenerative medicine. Here we highlight recent technical advances in CRISPR-based perturbomics, focusing on more physiologically relevant, single-cell-level analyses and their successful applications in discovering novel therapeutic strategies.

## CRISPR advances illuminate unannotated gene functions

Many genes in our genome are not well understood in terms of their function, even though they could be important for health and disease. This Review discusses how an approach called ‘perturbomics’ can help us learn about these lesser-known genes. Perturbomics involves changing a gene’s activity and observing the effects on cells. Researchers use a tool called CRISPR–Cas9, which can precisely edit genes, to perform these studies. CRISPR–Cas9 works by cutting DNA at specific points, which can disrupt a gene’s function. Scientists introduce these changes into cells and then observe how the cells behave under different conditions, such as drug treatments. This helps to identify which genes are important for certain traits or diseases. The findings from perturbomics can lead to new treatments for diseases such as cancer by identifying new drug targets.

This summary was initially drafted using artificial intelligence, then revised and fact-checked by the author.

## Introduction

Despite millions of studies published each year identifying gene–disease associations and causal relationships, a substantial proportion of gene products remain poorly characterized in terms of function^1^. Biological research tends to focus on well-characterized genes, with about 20% of protein-coding genes attracting nearly 95% of all published scientific literature about genes, largely due to the availability of genetic tools (for example, knockout mouse models, knockdown or overexpression constructs) and the ease of contextualizing their functions within existing signaling pathways or biological processes, making these studies more impactful. Meanwhile, studying the functions of lesser-characterized genes requires definitive evidence of their involvement in specific diseases or biological processes. Comparative genomics and transcriptomics can help to identify novel, uncharacterized genes associated with diseases, but these methods primarily establish associations, not causal relationships, and are often confounded by passenger mutations and differential expression with limited functional relevance. Functional genomics can overcome these limitations by directly annotating gene functions by uncovering their roles and interactions in biological processes, establishing causal links between genes and diseases. By adopting an unbiased approach, functional genomics has the potential to elucidate the functions of previously uncharacterized gene products, providing a foundation for novel therapeutic interventions.

Perturbomics is a functional genomics approach that annotates genes on the basis of the phenotypic changes induced by gene perturbation, typically through modulation of gene activity^2^. The central idea behind perturbomics is that the function of a gene product can best be inferred by altering its activity and systematically measuring the resulting phenotypic changes. The perturbomics approach was first applied more than two decades ago to identify genes that affect cell viability and invasion, using arrayed small interfering RNAs (siRNAs)—short, double-stranded RNA that mediate sequence-specific gene silencing— delivered to multiwell plates to assess phenotype changes^3^. However, early perturbomics screens had three major limitations: (1) off-target effects due to siRNAs degrading mRNAs with partial complementarity; (2) variability in siRNA efficiency, leading to potential false negatives due to incomplete gene knockdown; and (3) limited access to high-throughput facilities capable of handling large-scale multiwell assays. Two major technological advances addressed these challenges. First, the rapid development of massively parallel short-read sequencing enabled ‘pooled screens’, where gene disruption libraries are delivered as mixtures and phenotypic changes are assessed in a single sequencing run, eliminating the need for high-throughput facilities. Second, the advent of CRISPR–Cas9 technology^4^ allowed more precise, complete gene knockouts through frameshifting insertion or deletion (InDel) mutations, with fewer off-target effects than siRNAs. CRISPR screens, first performed around a decade ago, have since been used to identify core essential genes required for cell survival and genes that confer resistance to BRAF inhibitors, demonstrating the power of this approach in functional genomics and its ability to pinpoint genes whose perturbation induces specific phenotypic changes of interest^5^.

Over the past decade, important advances have enhanced the perturbomics approach, improving its robustness, accuracy and the diversity of phenotypes that can be analyzed. In particular, CRISPR screens combined with single-cell RNA sequencing (scRNA-seq) have shown great potential for comprehensive characterization of transcriptomic changes after gene perturbation. In addition, advances in organoid and stem cell technologies have enabled the study of therapeutic targets in more physiologically relevant, organ-mimetic systems. CRISPR screens have successfully identified novel therapeutic target genes whose modulation holds promise for treating a range of diseases, including cancer, cardiovascular diseases and neurodegenerative disorders. In this Review, we summarize recent technical progress in CRISPR-based perturbomics and highlight several successful case studies that have identified promising therapeutic target genes.

## Basic design of perturbomics study using CRISPR screens

**Fig. 1:**
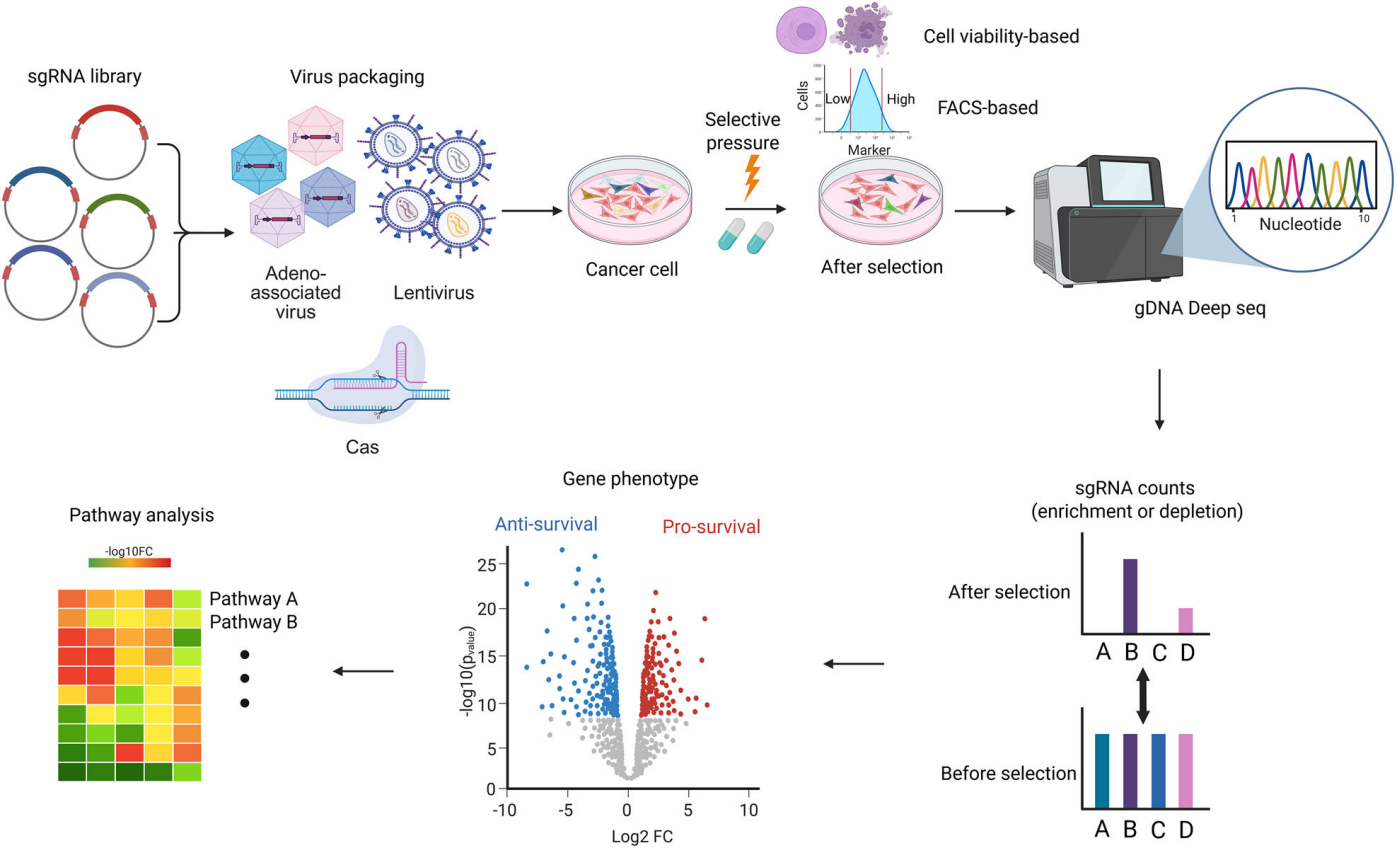
Schematic overview of the functional genomic screening workflow. The sgRNA library is used to generate viruses (adeno-associated virus or lentivirus) for packaging and delivery into cancer cells, with Cas9 expressed in target cells enabling targeted gene editing. After virus-mediated delivery, cells are subjected to selective pressures (for example, drugs, other stressors or FACS-based selection by marker expression). Genomic DNA (gDNA) is extracted, and sgRNA abundance is quantified using deep sequencing. Enrichment or depletion of sgRNAs is compared before and after selection, and differential counts are analyzed to determine gene phenotypes. Pathway analysis may be conducted to identify affected biological pathways.

The CRISPR–Cas9 system comprises two essential components: the Cas9 nuclease, which induces double-strand breaks in DNA, and the guide RNA (gRNA), which directs Cas9 to specific genomic loci. DNA cleavage by CRISPR–Cas9 is followed by DNA repair mechanisms, primarily nonhomologous end joining, which often introduces InDel mutations that result in frameshifts, effectively disrupting gene function. This property has facilitated the widespread use of CRISPR–Cas9-based knockout screens to efficiently identify genetic phenotypes. gRNA libraries, designed in silico to target either a genome-wide array of genes or specific gene sets, are synthesized as chemically modified oligonucleotides and cloned into a viral vector. The resulting viral gRNA library is transduced into a large population of Cas9-expressing cells, which are subsequently subjected to selective pressures. These pressures may include drug treatments or nutrient deprivation for cell viability screens, or fluorescence-activated cell sorting (FACS) to isolate cells expressing markers indicative of specific phenotypes. After selection, genomic DNA is extracted from the treated cell populations and analyzed using next-generation sequencing. The gRNAs present in the selected populations are amplified and sequenced to identify patterns of enrichment or depletion. The sequencing data are then processed using specialized computational tools to correlate specific genes with the observed phenotypes^6^. Positive hits from the initial screen are validated through follow-up experiments, such as individual gene knockouts or knockdowns, to confirm their functional relevance to the phenotype of interest. Further biological insights can be gained by investigating the roles of the identified genes in biological pathways, their molecular interactions and their potential therapeutic applications (Fig. 1).

## Technical advances in CRISPR screens

### Beyond loss-of-function screens

**Fig. 2:**
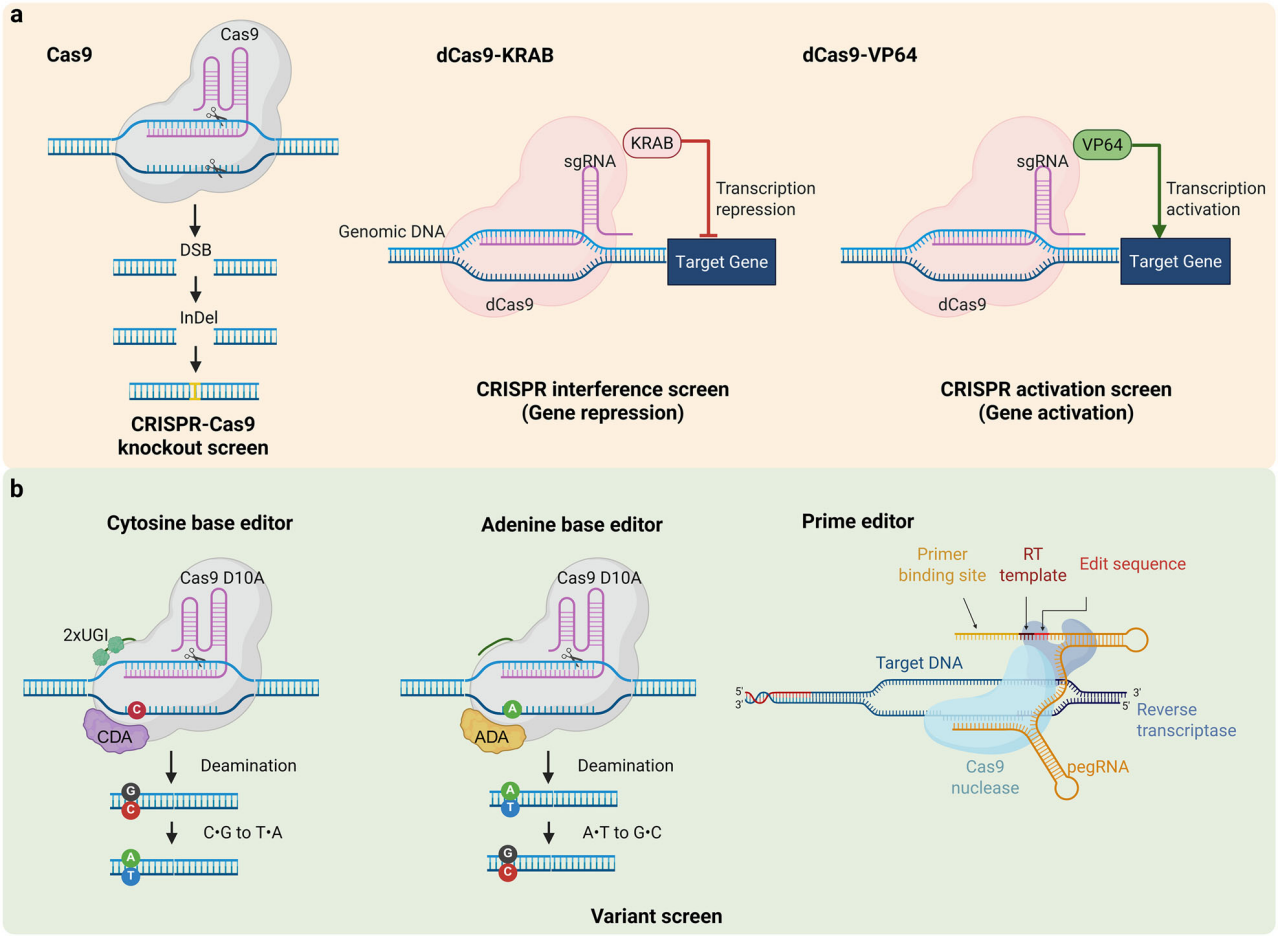
Overview of CRISPR screening with engineered Cas9. **a** The CRISPR–Cas9 knockout screen involves Cas9-mediated DNA double-strand breaks (DSBs) at target sites, leading to InDels that disrupt gene function. CRISPRi screens utilize catalytically inactive Cas9 (dCas9) fused with the KRAB repressor domain to repress target gene transcription. CRISPRa screens use dCas9 fused with the transcription activation domain such as VP64 to upregulate target gene expression. **b** Variant screens by base editing engineered Cas9: cytosine base editors that combine a Cas9 D10A mutant nickase, a cytidine deaminase (CDA) and uracil glycosylase inhibitors (UGIs) to convert cytosine (C) to thymine (T), resulting in a C•G-to-T•A substitution. Adenine base editors utilize a Cas9 D10A mutant nickase and adenine deaminase (ADA) to convert adenine (A) to guanine (G), achieving an A•T-to-G•C substitution. Prime editors integrate a Cas9 nickase with reverse transcriptase and a prime editing guide RNA (pegRNA) containing a target site, reverse transcriptase (RT) template and edit sequence to introduce precise DNA modifications.

While knockout screens using Cas9 have been invaluable, they are not without limitations (Fig. 2a). Cas9-mediated gene ablation introduces random InDel mutations that lead to frameshifts, limiting its use to protein-coding genes with reading frames. This precludes the application of knockout screens to long noncoding RNAs (lncRNAs). In addition, DNA double-strand breaks induced by Cas9 are inherently toxic. Accordingly, the viability of cells upon gene ablation is strongly influenced by the copy number of the targeted gene, which correlates with the number of double-strand breaks by a gRNA^7^. Finally, to improve the reliability of screening hits, loss-of-function screens can be combined with gain-of-function screens, enabling better prioritization of candidates for further validation.

The modular nature of Cas9 function, binding and cleaving the target DNA, allows it to be repurposed for both loss-of-function and gain-of-function studies. For example, *Streptococcus pyogenes* Cas9 (SpCas9) has two nuclease domains, RuvC and HNH, responsible for cleaving each DNA strand. Mutating key catalytic residues (D10 in RuvC and H840 in HNH) generates a nuclease-inactive Cas9 (dCas9) that retains its RNA-guided DNA binding ability^4^. By fusing dCas9 to functional domains, researchers can modulate gene expression at specific genomic loci. For instance, dCas9 fused to the KRAB transcriptional repressor silences genes, while activators such as VP64, VP64-p65-Rta (VPR) or synergistic activation mediator (SAM) enable gene activation^8^ (Fig. 2a). With the advent of RNA-guided RNA cleaving CRISPR–Cas13 (ref. ^9^), knockdown screens involving specific degradation of mRNA are also emerging. This approach has facilitated both loss-of-function and gain-of-function screens. dCas9–KRAB-mediated knockdown (CRISPR interference, CRISPRi) screens may complement Cas9-mediated knockout screens by enabling the targeting of lncRNAs and transcriptional enhancer elements and by enabling the loss-of-function screens in cells that are particularly sensitive to DNA double-strand breaks, such as embryonic stem (ES) cells^10^. Meanwhile, dCas9-activator-mediated gain-of-function (CRISPR activation, CRISPRa) screens complement loss-of-function studies by enhancing the confidence in identifying target genes.

CRISPR screens have also been adapted for variant studies, enabling the functional analysis of genetic variants^11^ (Fig. 2b). Base editors, which tether enzymatic domains to a nuclease-impaired Cas9, allow precise nucleotide modifications. For example, cytidine deaminase enables cytosine-to-thymine transitions (cytidine base editing), while an evolved *Escherichia coli TadA* facilitates adenine-to-guanine transitions (adenine base editing)^12^. Reverse transcriptase enzymes have also been used in prime editors to induce small-scale insertions, deletions or substitutions^12^. These tools, when combined with CRISPR screens, enable the generation of libraries of point mutant variants for high-throughput functional annotation. This approach has been instrumental in identifying the functional relevance of single-nucleotide variants of unknown significance. For example, Kim and colleagues used a prime-editor-based tiling array of over 2,000 single-nucleotide variants in epidermal growth factor receptor (EGFR) to functionally evaluate their ability to induce resistance against EGFR inhibitors^13^. Despite its utility, base and prime editor screens are limited to generating single, small-sized mutations within a specific editing window defined by the protospacer adjacent motif (PAM). This restricts the diversity of mutants and prevents the study of continuous evolution involving multiple mutations. To address these limitations, base editors have been tethered to processive enzymes such as T7 RNA polymerase or DNA helicase, enabling continuous evolution in mammalian cells. Platforms like TRACE (T7 polymerase-driven continuous editing)^14^ tether base editors to T7 RNA polymerase, facilitating continuous editing of a target locus downstream of a T7 promoter. This overcomes PAM restrictions and broadens the scope of base editing. Using these systems, researchers identified MEK1 variants conferring resistance to MEK1 inhibitors, respectively. The HACE system (Cas9 nickase tethered to DNA helicase)^15^ has further expanded the potential for continuous evolution by enabling the editing of specific endogenous genes. This approach has uncovered MEK1 inhibitor resistance mutations and SF3B1 variants linked to RNA splicing. These advances summarized in Table 1 demonstrate the potential of continuous evolution platforms to address the limitations of traditional CRISPR screening methods.

**Table 1:** Technical advances in functional genomics screens.

Name	Notable advances	Reference
PEER-seq (prime editing and endogenous region sequencing)	Prime-editor-based, massively parallel generation of single-nucleotide variants for functional annotation of variants of uncertain significance	^13^
TRACE (T7 polymerase-driven continuous editing)	Continuous editing of exogenous gene for identification of gene mutations that alter gene function	^14^
HACE (helicase-assisted continuous editing)	Continuous editing of endogenous gene for identification of gene mutations that alter gene function	^15^
Perturb-Seq	CRISPR screens with single-cell level transcriptome as readout	^16^
CROP-seq (CRISPR droplet sequencing)		^17^
CRISP-seq		^18^

### Diversifying readouts for CRISPR screens

Conventional CRISPR screens primarily rely on massively parallel sequencing of PCR amplicons to monitor changes in the relative abundance of single guide RNAs (sgRNAs)—short RNA that guide the CRISPR-Cas9 complex to specific genomic loci— within a bulk cell population subjected to selection pressure or sorted on the basis of a specific surrogate marker. As a result, the readouts of these screens are generally restricted to metrics such as cell viability or simple protein marker expression that can be analyzed using FACS. This limitation constrains the ability to investigate complex transcriptional profiles and the intricate interactions within cellular pathways at high resolution. To overcome this restriction, approaches have been developed that integrate pooled CRISPR screens with scRNA-seq, such as Perturb-seq^16^ and CROP-seq^17^. These methodologies enable detailed transcriptional profiling of cells with specific gene knockouts, offering a more comprehensive understanding of gene functions and regulatory networks. For instance, CRISP-seq^18^ has been used to reconstruct gene regulatory circuits for transcription factors (TFs) in bone marrow-derived dendritic cells. Perturbation of TFs revealed distinct regulatory modules, including M1 (Stat1 and Stat2), M2 (Cebpb, JunB, Rela, Stat3 and Hif1), M3 (Rel, Irf2 and Atf3) and M4 (Spi1, Runx1, Irf4 and Nfkb1). Similarly, CROP-seq in T cells demonstrated that knockout of *LCK* or *ZAP70* produced transcriptomic profiles resembling those of naive T cells, effectively suppressing T cell receptor activation. These advanced screening techniques enhance the resolution and breadth of CRISPR-based functional studies, enabling the exploration of complex cellular responses and regulatory networks with unprecedented detail.

## Identification of novel cancer therapeutic strategies using perturbomics

Perturbomics studies, particularly those leveraging CRISPR screens, have emerged as powerful tools in cancer research. These studies enable the identification of therapeutic insights by linking changes in cell viability to underlying genetic perturbations. Landmark projects such as the DepMap project^19^ have generated extensive datasets of genome-wide CRISPR screening results across diverse cancer cell lines. Beyond standard viability screens in typical culture conditions, perturbomics approaches are increasingly applied to contexts such as tumor immune response and nutrient limitation. These efforts aim to uncover therapeutic targets that enhance tumor immunity or exploit cancer-specific metabolic vulnerabilities. This section highlights key findings and potential therapeutic targets revealed through CRISPR screens in cancer research. Key experimental strategies and target genes identified as potential therapeutic targets against cancer discussed below are summarized in Table 2.

**Table 2:** Perturbomics studies identifying therapeutic genes targeting cancer.

Targets for druggable vulnerabilities				
Gene(s) identified	Screening system	Experimental model subject to CRISPR screen	Drug treated	Reference
*WRN*	CRISPR–Cas9 KO	Multiple cell lines		^22^
*CIP2A*	CRISPR–Cas9 KO	RPE1–hTERT (BRCA1^−/−^) and DLD1 (BRCA2^−/−^)		^23^
*PKMYT1*	CRISPR–Cas9 KO	RPE1–hTERT (CCNE1 OE)		^24^
*FAM50A/FAM50B*	CRISPR–Cas9 KO	A375 and MeWo		^26^
*FYN*	CRISPR–Cas9 KO	MDA-MB-231	NVP-ADW742, gefitinib and imatinib	^27^
*AMD1*	CRISPR–Cas9 KO	A375 VR	Vemurafenib	^29^
*CDK6*	CRISPR–Cas9 KO	M238R1	PLX4720	^30^
*SHOC2*	CRISPR–Cas9 KO	CFPAC-1, A549 and NCH-h23	Trametinib	^31^
GRB7-mediated RTK pathway	CRISPR–Cas9 KO	HCT116	AZD6244	^32^
ILK–GSK3B pathway	CRISPR–Cas9 KO	KatoIII	AZD4547	^33^
*EGFR*	CRISPR–Cas9 KO	SNU449	Lenvatinib	^34^
*PRMT5*	CRISPR–Cas9 KO	In vivo (PDX model)	Gemcitabine	^35^
*HNRNPU*	CRISPR–Cas9 KO	T24	Cisplatin	^36^
*PLK4*	CRISPR–Cas9 KO	HCT116, DLD1, T47D, MCF7, A549 and NCI-H1568	Oxaliplatin	^37^
*RNASEH2B*	CRISPR–Cas9 KO	Sum149pt, HeLa and RPE1–hTERT	Olaparib and talazoparib	^38^
*MMS22L* and *CHEK2*	CRISPR–Cas9 KO	PCa, LNCaP, C4-2B, 22Rv1 and DU145	Olaparib	^39^
**Cancer immunotherapy targets**				
**Gene(s) identified**	**Screening system**	**Experimental model subject to CRISPR screen**	**Reference**	
*APLNR*	CRISPR–Cas9 KO	Mel624–T cell coculture	^42^	
*TRAF3*	CRISPR–Cas9 KO	B16F10–T cell coculture	^43^	
*PTPN2*	CRISPR–Cas9 KO	B16F10 syngeneic graft	^44^	
*ADAR1*	CRISPR–Cas9 KO	B16F10 syngeneic graft	^45^	
*SETDB1*	CRISPR–Cas9 KO	B16F10/LLC syngeneic graft	^46^	
*Ado*	CRISPR–Cas9 KO	MC38-Ova and OT-I T cell syngeneic graft	^47^	
*KDM3A*, *KLF5*, *SMAD4* and *EGFR*	CRISPR–Cas9 KO	Murine Kras/p53 mutant pancreatic ductal adenocarcinoma cell graft	^48^	
*Asf1a*	CRISPR–Cas9 KO	Murine Kras/p53 mutant lung adenocarcinoma cell graft	^49^	
*Cop1*	CRISPR–Cas9 KO	4T1 syngeneic graft	^50^	
*Lgals2*	CRISPR–Cas9 KO	4T1 syngeneic graft	^51^	
*Serpinb9* and *Adam2*	CRISPR–Cas9 KO	LSL-Kras^G12D^ or LSL-Braf^V600E^ mice	^52^	
*Dhx37*	CRISPR–Cas9 KO	Adoptively transferred OT-I T cells in E0771 tumor-bearing mice	^53^	
*PRODH2*	Dead-guide RNA (dgRNA)-based CRISPRa screen	OT-I T cells cocultured with E0771	^54^	
*CALHM2*	AAV–CRISPR–Cas9 KO	Adoptively transferred NK cells	^55^	
*Pofut1*	CRISPR–Cas9 KO	Adoptively transferred T cells in LCMV-Arm infection model mice	^56^	
*Flcn*	CRISPR–Cas9 KO	Adoptively transferred OT-I T cells in *Listeria monocytogenes*-OVA infection model mice	^57^	
*SEC31A*	CRISPR–Cas9 KO	in vitro primed mouse primary T_reg_ cells	^58^	
*BATF3*	CRISPRi/CRISPRaCRISPR–Cas9 KO	Human primary CD8 T cells	^59^	
*PIK3CD*, *VAV1*, *LCP2*, *PLCG1* and *DGKZ*	Base editing screen	Human primary T cells	^60^	
**Targets for metabolic vulnerabilities**				
**Gene(s) identified**	**Screening system**	**Experimental model subject to CRISPR screen**	**Related metabolic pathways**	**Reference**
*METTL16*	CRISPR–Cas9 KO	NB4	Amino acid metabolism	^61^
*ASNS*	CRISPR–Cas9 KO	Depmap data analysis		^62^
*GPT2*	CRISPR–Cas9 KO	K562		^63^
Amino acid transporters	CRISPRi and CRISPRa	K562		^64^
*NGRN*	CRISPR–Cas9 KO	K562		^65^
*KEAP1*	CRISPR–Cas9 KO	MDA-MB-231		^66^
*AMD1*	CRISPR–Cas9 KO	A375	Polyamine metabolism and OXPHOS	^29^
*CDK9*, *DHODH* and *PRMT5*	CRISPR–Cas9 KO	MOLM-13	OXPHOS	^68^
*PEX3* and *PEX10*	CRISPR–Cas9 KO	OVCAR-8 and 786-O	Ferroptosis	^69^
*PKCβII*	CRISPR–Cas9 KO	MDA-MB-231		^70^
*MBOAT1/2*	CRISPR–Cas9 KO	HT1080		^71^
*PSTK*	CRISPR–Cas9 KO	Hep3B and SNU-398		^72^
*DHCR7*	CRISPR–Cas9 KO	Pfa1		^73^

*KO* knockout.

### Identifying druggable vulnerabilities in undruggable tumors

**Fig. 3:**
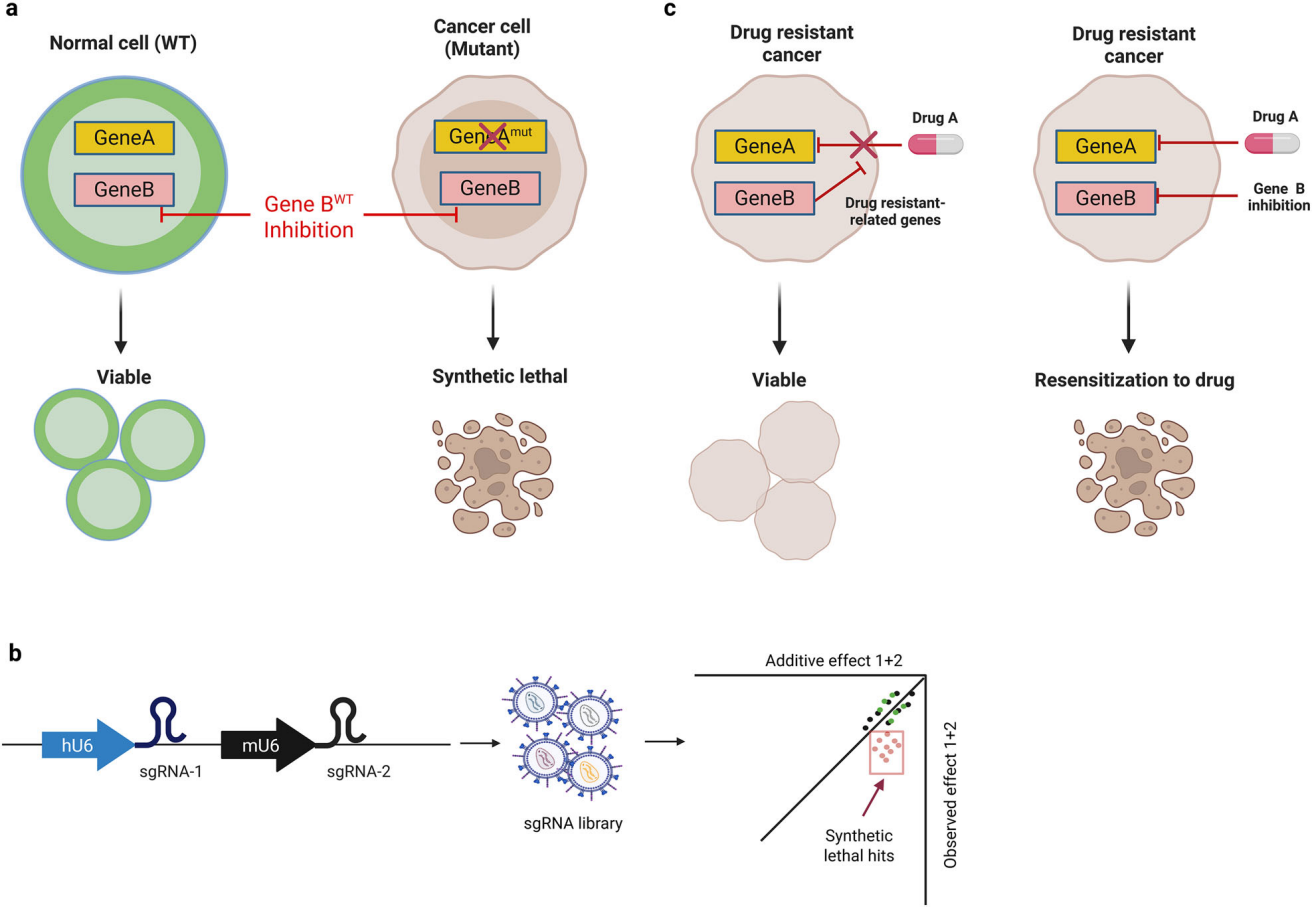
Perturbomics approach to identify druggable targets against undruggable cancer. **a** Concept of synthetic lethality: in normal cells (wild type), inhibition of gene B does not affect cell viability. However, in cancer cells with a mutated gene A (gene A^mut^), the inhibition of gene B leads to synthetic lethality, resulting in cancer cell death. **b** Combinatorial CRISPR screen identifying synthetic lethality: a dual- (or higher-order) sgRNA library is used to target combinations of genes simultaneously. The synthetic lethality effect is assessed by comparing the observed combined effects to the additive effects of individual gene disruptions. **c** Targeting drug-resistant cancer: perturbomics can be used to identify secondary genes (gene B) whose modulation resensitizes drug-resistant cancer cells to drug A, resulting in cell death and offering a potential therapeutic strategy.

Targeted therapies have revolutionized cancer treatment, with successes such as HER2 inhibition in HER2-amplified breast cancer and EGFR-targeted therapies in EGFR-mutated lung cancer. However, many cancers lack clearly druggable targets. For example, cancers often exhibit mutations in hard-to-drug oncogenes such as KRAS^20^ or loss of tumor suppressors such as PTEN^21^, creating an urgent need for novel therapeutic strategies. One promising approach is synthetic lethality, where the simultaneous perturbation of two genes leads to cancer-specific cell death (Fig. 3a). CRISPR screens have been instrumental in identifying synthetic lethal interactions, such as WRN helicase in microsatellite instability-high colorectal cancer^22^. Similarly, targeting the CIP2A–TOPBP1 axis was shown to impair genome stability in *BRCA*-mutant tumors^23^. PKMYT1 inhibition has also demonstrated synthetic lethality in *CCNE1*-amplified tumors, with pharmacological inhibition synergizing effectively with gemcitabine^24^.

Combinatorial CRISPR screening has further expanded the ability to uncover synthetic lethal interactions (Fig. 3b). Conventional CRISPR screens operate on the premise that each transduced cell integrates only a single sgRNA. As a result, they cannot analyze phenotypic changes caused by the simultaneous perturbation of multiple genes, making it difficult to uncover novel gene interactions or effectively target closely related paralogs with redundant functions. To address these limitations, many groups have developed a combinatorial CRISPR screen workflow involving gRNA library containing multiple gRNAs in each plasmid^25^. For instance, Adams et al. identified the FAM50A–FAM50B gene pair as synthetic lethal in melanoma^26^. Moreover, combinatorial CRISPR screens targeting tyrosine kinases in triple-negative breast cancer (TNBC) cells revealed that FYN disruption synergizes with inhibition of IGF1R, EGFR and ABL, suggesting potential for combinatorial therapies using tyrosine kinase inhibitors^27^.

Receptor tyrosine kinases (RTKs) and the downstream MAPK pathway are frequent drivers of cancer and primary targets for inhibitors of BRAF, MEK1 and ERK1. However, resistance often develops due to compensatory activation of alternative pathways, such as PI3K signaling, or metabolic adaptations^28^. CRISPR screens have been critical in uncovering resistance mechanisms (Fig. 3c). For instance, we performed CRISPR screens in vemurafenib-resistant melanoma cells to identify *S*-adenosylmethionine decarboxylase (AMD1) as a mediator of resistance through enhanced polyamine biosynthesis^29^. Persistent c-Myc activation, downstream of MAPK and PI3K–AKT signaling, was found to drive polyamine biosynthesis and subsequent resistance, highlighting a critical link between signal transduction and metabolic adaptation in BRAF inhibitor resistance. Similarly, CDK6 was identified as a driver of resistance in melanoma^30^. Resistance to MEK inhibitors has also been attributed to proteins such as SHOC2^31^ and GRB7^32^ in cancers with mutant RAS. In addition, CRISPR screens revealed the ILK–GSK3B axis as a resistance mechanism against FGFR inhibitors in gastric cancer^33^. The combination of lenvatinib and gefitinib was found to synergistically target multiple tyrosine kinases in hepatocellular carcinoma^34^.

DNA-damaging chemotherapeutics remain standard treatments for many cancers, although resistance frequently arises. CRISPR screens have identified genes whose loss sensitizes cancer cells to these agents. For example, PRMT5 loss sensitized pancreatic cancer cells to gemcitabine by impairing DNA repair^35^. Similarly, depletion of HNRNPU and PLK4 sensitized cells to cisplatin and oxaliplatin, respectively^36,37^. Targeted therapies such as PARP inhibitors (for example, olaparib) exploit vulnerabilities in DNA damage response pathways, particularly in BRCA1/2-deficient tumors. However, resistance can emerge via alternative DNA repair pathway activation. CRISPR screens have identified genes such as RNASEH2B whose loss enhances PARP inhibitor sensitivity by impairing nucleotide excision repair^38^. In addition, MMS22L loss increases olaparib sensitivity in prostate cancer by disrupting RAD51-mediated homologous recombination, while CHEK2 loss confers resistance through BRCA2 upregulation^39^. Combining ATR and PARP inhibitors has also shown efficacy in overcoming such resistance^39^.

### Identifying novel cancer immunotherapy targets

**Fig. 4:**
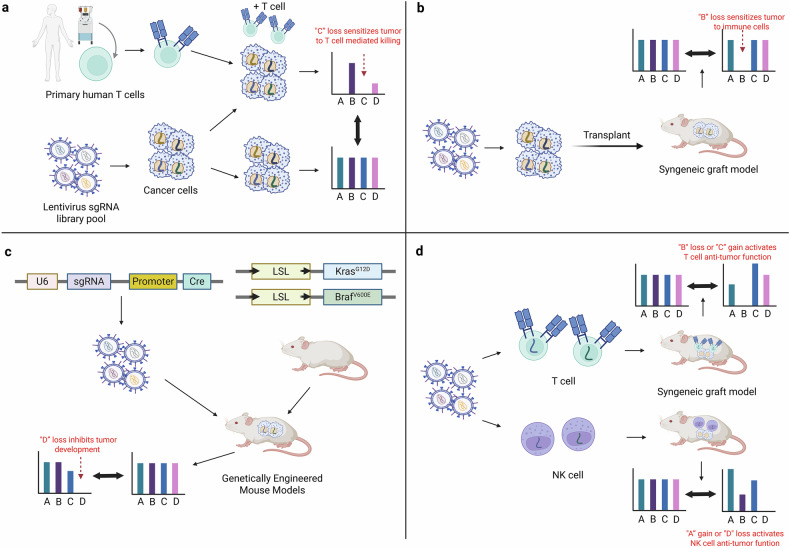
Perturbomics approach to identify cancer immunotherapy targets. **a** CRISPR screens in cancer cells cocultured with immune cells (for example, primary human T cells) can identify genes whose loss can sensitize cancer to T-cell-mediated killing. **b** Cancer cells delivered with sgRNA library can be subjected to syngeneic graft experiment to discover genes that modulate cellular fitness in vivo. **c** In vivo CRISPR screens are done in genetically engineered mouse models of cancer to fully model the TME. **d** CRISPR screens are done with primary T cells or NK cells to find genes that modulate immune cell activity in tumors.

The success of immune checkpoint inhibitors (ICIs) and chimeric antigen receptor (CAR) T cell therapies has catalyzed tremendous interest in cancer immunotherapy. Despite these advances, substantial limitations persist. Most patients treated with ICIs show limited or no benefit^40^, and while CAR T cell therapy has shown durable efficacy in hematologic malignancies such as B cell cancers, it has largely failed in solid tumors^41^. A major barrier to these therapies is the immune-suppressive tumor microenvironment (TME), which dampens antitumor immune responses. CRISPR screens have emerged as a powerful tool to identify cancer cell-intrinsic factors driving immune evasion and the development of immune-suppressive TMEs (Fig. 4a). Initial CRISPR screens for immunotherapy targets were performed in tumor–T cell coculture systems. For example, Restifo and colleagues, using NY-ESO-1 as a model antigen, identified *APLNR* as a gene whose loss sensitizes cancer cells to T-cell-mediated killing^42^. Liu et al. screened for genes regulating PD-L1 or MHC-I expression under interferon-γ treatment in a B16F10 melanoma model and identified TRAF3 as a key suppressor of NF-κB signaling and MHC-I antigen presentation^43^.

To model more complex cancer–immune cell interactions, in vivo CRISPR screens with syngeneic tumor grafts have been developed (Fig. 4b). Using B16F10 melanoma models, Haining and colleagues identified PTPN2, ADAR1 and SETDB1 as regulators of the JAK–STAT pathway, double-stranded RNA sensing and endogenous viral antigen silencing, respectively^44–46^. Using MC38 colon cancer model in vitro (coculture with T cells) and in vivo (cotransplantation of MC38-Ova cells and OT-I T cells), Oliaro and colleagues found that deletion of 2-aminoethanethiol dioxygenase (Ado) sensitized tumors to TNF-mediated cytotoxicity^47^. Syngeneic models treated with chemotherapy and ICIs have identified genes such as KDM3A that sensitized pancreatic ductal adenocarcinoma to immunotherapy^48^. Studies also revealed regulators of tumor–macrophage crosstalk, including Asf1a, Cop1 and Lgals2, whose loss improved immune responses in TNBC and lung cancer models^49–51^. Although syngeneic grafts provide valuable insights, they do not perfectly recapitulate the TME. Genetically engineered mouse models have been used as an alternative (Fig. 4c). Using *LSL-Kras*^*G12D*^ or *LSL-Braf*^V600E^ models with lentiviral sgRNA libraries, Serpinb9 was identified as a key immune evasion factor in lung cancer^52^.

CRISPR screens have also been used to identify genes intrinsic to immune cells, such as T cells and natural killer (NK) cells, that enhance their activity in immune-suppressive TMEs (Fig. 4d). Chen and colleagues used a genome-wide knockout sgRNA library in primary CD8^+^ T cells from Cas9-transgenic mice, identifying Dhx37, which modulates CD8^+^ T cell function via NF-κB signaling^53^. Using a CRISPRa library, the same group found that PRODH2 reprograms proline metabolism to enhance T cell activity^54^. They also used an adeno-associated virus (AAV)-packaged sgRNA library to reveal CALHM2 as a key NK cell activity regulator^55^.

CRISPR screens have also elucidated mechanisms regulating immune cell fate, with potential implications for tumor immunity. For example, in a lymphocytic choriomeningitis virus (LCMV) infection model, Pofut1 was found to regulate the differentiation of effector and memory T cells via mTORC1 signaling^56^. Similarly, Flcn enhanced tissue-resident memory T cell function in a *Listeria monocytogenes* model^57^. In another study, using mouse primary T cells in vitro primed for regulatory T cell differentiation, SEC31A was identified as suppressor of T cell differentiation into regulatory T cells^58^. CRISPRa and CRISPRi screens in human primary T cells identified BATF3 as an epigenetic regulator of memory T cell formation, controlling genes such as IL7R^59^. Marson and colleagues recently conducted a tiling CRISPR screen to identify point mutations in genes such as *PIK3CD*, *VAV1*, *LCP2*, *PLCG1* and *DGKZ* that regulate CD8^+^ T cell activity^60^. Advances in near-PAM-less Cas9 systems may enable more comprehensive analysis of critical residues in immune-modulating genes.

### Discovery of novel metabolic vulnerabilities in cancer

Cancer cells display distinct metabolic adaptations compared with normal cells, including alterations in glycolysis, oxidative phosphorylation (OXPHOS), amino acid metabolism, polyamine metabolism and lipid metabolism. These differences present opportunities to exploit cancer-specific metabolic vulnerabilities for therapeutic purposes. Perturbomics, a technique combining functional genomics with metabolic analysis, has become instrumental in identifying metabolic pathways critical for cancer survival. Accordingly, genome-wide CRISPR screens have revealed metabolic vulnerabilities across various cancer models. For instance, in NB4 acute myeloid leukemia cells, METTL16 was identified as a regulator of branched-chain amino acid metabolism, essential for leukemia-initiating cells^61^. By integrating metabolomics data from nearly 1,000 cancer cell lines with DepMap datasets, Sellers and colleagues identified asparagine synthetase as a critical vulnerability in asparagine-enriched cancer cell lines^62^.

Traditional tissue culture media, often rich in nutrients, do not accurately mimic physiological conditions, especially the nutrient-deprived TME. To address this, researchers have performed CRISPR screens in media that simulate physiological conditions or impose specific nutrient limitations. For example, Cantor and colleagues used the K562 chronic myeloid leukemia cell line cultured in classical RPMI1640 or physiologic human plasma-like medium, which mirrors average nutrient concentrations in healthy adults, revealing dependencies on alanine and pyruvate for tumor progression^63^. Chidley et al. identified amino acid transporters as therapeutic targets in chronic myeloid leukemia using amino-acid-depleted media^64^. In addition, by substituting glucose with galactose, which selectively kills OXPHOS-deficient cells, Mootha and colleagues pinpointed genes such as NGRN as essential for OXPHOS^65^.

Therapy-resistant cancers often exhibit metabolic rewiring, making perturbomics a valuable approach to identify novel vulnerabilities. Weyemi and colleagues performed CRISPR screens in MDA-MB-231 TNBC cells treated with the ATM inhibitor AZD1390, uncovering that *KEAP1* disruption synergized with ATM inhibition^66^. Upregulation of OXPHOS is observed in some tumors and may be associated with therapy resistance^67^. We recently demonstrated that inhibiting AMD1, a key enzyme in polyamine biosynthesis, enhanced sensitivity to BRAF inhibitors by suppressing polyamine synthesis and EIF5A hypusination, ultimately downregulating OXPHOS^29^. Similarly, Lapalombella and colleagues identified CDK9, DHODH and PRMT5 as targets to overcome resistance to the FLT3 inhibitor gilteritinib in acute myeloid leukemia by blocking OXPHOS^68^.

Ferroptosis, an iron-dependent form of cell death characterized by the accumulation of lipid peroxides, has emerged as a promising therapeutic target. Cancer cells evade ferroptosis through antioxidant mechanisms involving cysteine transport, the GSH–GPX4 pathway or the CoQ-dependent FSP1 system. CRISPR screens have identified numerous ferroptosis regulators. For example, the peroxisomal genes PEX3 and PEX10 were shown to regulate ferroptosis by mediating the synthesis of polyunsaturated ether phospholipids (PUFA-ePLs)^69^, a source for lipid peroxidation that drives ferroptosis. In TNBC, PKCβII promoted ferroptosis by enhancing lipid peroxidation^70^. Hormonal signaling was implicated in ferroptosis regulation in breast and prostate cancers, with MBOAT1 and MBOAT2 modulating phospholipid composition to suppress ferroptosis^71^. In hepatocellular carcinoma, PSTK negatively regulated the GSH–GPX4 pathway by inhibiting cysteine synthesis^72^. Freitas et al. had also identified using CRISPR screens with ferroptosis model cell line Pfa1 to DHCR7, a cholesterol biosynthesis gene, as a regulator of ferroptosis by reducing levels of 7-dehydrocholesterol, which protects lipids from lipid peroxidation^73^.

## Perturbomics for deciphering cell fate and differentiation

**Fig. 5:**
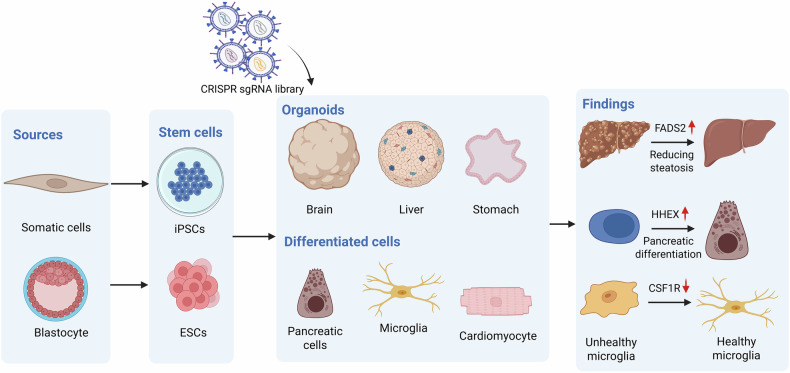
Perturbomics for deciphering cell fate and differentiation. Perturbomics approaches are applied in various contexts by delivering sgRNA libraries to iPS cells, ES cells or organoids, and in vitro differentiating to specific cellular lineages to find genes that regulate pathological conditions such as steatosis in hepatocyte as well as cellular differentiation.

As briefly mentioned with CRISPR screens used to identify key regulators of immune cell fate, perturbomics offers a powerful approach to unravel the mechanisms governing cell fate decisions. Studies leveraging stem cells, their differentiated progenies, organoids and in vivo mouse models have illuminated molecular mechanisms underlying cellular differentiation, providing insights into development and the pathogenesis of diseases such as neurodegeneration. These findings highlight the potential of perturbomics to identify regulatory genes across organs, potentially advancing targeted therapies in regenerative medicine (Fig. 5). Key experimental strategies and target genes identified as cell fate regulators discussed below are summarized in Table 3.

**Table 3:** Perturbomics studies identifying genes regulating cell fate and differentiation.

Organ	Gene(s) identified	Screening system	Experimental model subject to CRISPR screen	Reference
Brain	*PSAP*	CRISPRi and CRISPRa	Human iPS cell-derived glutamatergic neuron	^74^
	IL-6 and NF-κB pathway	CRISPRi + scRNA-seq	Human iPS cell-derived astrocyte	^75^
	*UBA3*	CRISPR–Cas9 KO	Alzheimer’s disease stem cell	^76^
	*GRN*	CRISPR–Cas9 KO	BV-2 microglial cell line	^77^
	*SEC24B*	CRISPR–Cas9 KO	Human immortalized microgial cell line	^78^
	*CSF1R*, *CDK12*, *MED1* and *INPP5D*	CRISPRi/CRISPRa	Microglia	^79^
Heart	*RNF20/40*	AAV–CRISPR–Cas9 KO	In vivo (mice)	^80^
	*PRELP* and *JAZF1*	CRISPR–Cas9 KO	Human cardiac fbroblasts	^81^
	*NF2*	CRISPR–Cas9 KO	Human iES cell-derived cardiomyocyte	^82^
	*BRD4*	CRISPR–Cas9 KO	Human iPS cell-derived cardiomyocyte	^83^
Liver	*BAZ2*	CRISPR–Cas9 KO	Fah-knockout mouse model	^84^
	*SPP2*	CRISPR–Cas9 KO		^85^
	*MIER1*	CRISPR–Cas9 KO		^86^
	*FADS2*	CRISPR–Cas9 KO	Hepatic organoids	^87^
	*Fos* and *Ubr5*	CRISPR–Cas9 KO	Intrahepatic cholangiocyte organoids	^88^
Kidney	*ROCK1*	CRISPR–Cas9 KO	Kidney organoids	^89^
Intestine	*SMARCA4* and *SMARCC1*	CRISPR–Cas9 KO	Fetal/intestinal organoids	^90^
Stomach	*Alk*, *Bclaf3* and *Prkra*	CRISPR–Cas9 KO	Stomach organoids	^91^
Pancreas	*HHEX*	CRISPR–Cas9 KO	iPS cell-derived pancreatic lineage differentiation model	^92^

*KO* knockout.

### Perturbomics study for understanding neurodevelopmental and neurodegenerative diseases

Neurodegenerative diseases such as Alzheimer’s and Parkinson’s are rising in prevalence in aging populations, yet their underlying mechanisms remain elusive. Perturbomics studies in this domain are complex owing to the lack of easily scalable and manipulable models like cancer cell lines. Instead, many perturbomics studies are relying on ES cells or induced pluripotent stem (iPS) cells differentiated into disease-relevant cell types. For instance, Kampmann’s group developed iPS cell lines stably expressing CRISPRi and CRISPRa systems to generate neurons, astrocytes and microglia. Using this platform, they identified therapeutic targets for Alzheimer’s disease, including PSAP (a neuronal ferroptosis modulator)^74^ and pathways such as IL-6 signaling and NF-κB as key mechanisms in Alzheimer’s and hypoxic–ischemic encephalopathy^75^. Similarly, studies using isogenic iPS cell pairs with and without amyloid beta accumulating *APP*^*swe/swe*^ mutation found UBA3, a subunit of E1 ligase, as essential in Alzheimer’s neurons^76^.

CRISPR screens have also explored microglial function critical to brain immunity. Using the murine BV-2 microglial cell line, progranulin (GRN) was identified as a regulator of microglial activity and lipid droplet accumulation in neurodegenerative diseases^77^. In immortalized human microglial cells, SEC24B emerged as a key regulator of ferroptosis in microglia^78^. Kampmann’s group, using an approach similar to the one mentioned above, utilized iPS cell-derived microglial cells to identify CSF1R as critical mediator of microglial survival, CDK12 and MED1 for microglial activity, and INPP5D for phagocytosis^79^.

### Perturbomics study in cardiomyocyte differentiation

Perturbomics has also advanced our understanding of cardiomyocyte differentiation and maturation. Perinatal delivery of AAV-packaged CRISPR libraries in mice in vivo revealed RNF20 and RNF40 as transcriptional regulators of cardiomyocyte maturation^80^. Ellinor’s group conducted arrayed CRISPR screens with high-content imaging in human cardiac fibroblasts, identifying PRELP and JAZF1 as modulators of fibroblast-to-myofibroblast transition, a process linked to cardiomyopathy^81^. In vitro CRISPR screens in the context of cardiomyocyte differentiation of ES and iPS cell differentiation pinpointed NF2 as a regulator of epithelial–mesenchymal transition into mesodermal lineages and BRD4 as an epigenetic modulator of cardiomyocyte differentiation genes^82,83^.

### Perturbomics in epithelial tissues for studying tissue regeneration and differentiation

The liver’s remarkable regenerative capacity—capable of recovering from up to 80% hepatectomy—makes it a prime model for studying tissue regeneration. Efficient delivery of gRNA libraries via tail vein AAVS injection or hydrodynamic injection enables in vivo CRISPR screens. In the fumarylacetoacetate hydrolase (Fah)-knockout mouse model, gRNA libraries codelivered with Fah gene allow hepatocytes expressing Fah (and gRNA) to dominate and regenerate the liver upon withdrawal of NTBC (2-(2-nitro-4-trifluoromethylbenzoyl)-1,3-cyclohexanedione), a drug that prevent liver damage upon Fah loss induced tyrosinemia by inhibiting tyrosine catabolism. This platform provides an optimal model for discovering genes that influence regenerative capacity of hepatocytes and was successfully applied to identify BAZ2^84^, SPP2^85^ and MIER1^86^ as negative regulators of hepatic regeneration. For nonalcoholic fatty liver disease, Clevers and colleagues used hepatic organoid cultures with high-content imaging to score steatosis, identifying FADS2 as a key regulator of lipid accumulation via a lipogenesis-dependent mechanism^87^. Wang and colleagues performed CRISPR screens with primary intrahepatic cholangiocyte organoids and performed scRNA-seq analysis to identify identified Fos and Ubr5 as mediators of hepatocyte differentiation and maturation^88^.

Organoids are widely used to investigate epithelial tissue differentiation and fate determination in other epithelial organs. In kidney organoids derived from iPS cells, CRISPR screens identified ROCK1 as a critical regulator of early differentiation, shedding light on kidney development and chronic kidney disease^89^. Similarly, in fetal intestinal organoids, SMARCA4 and SMARCC1 were found to modulate epithelial maturation^90^. In stomach organoids, a CRISPR screen revealed Alk, Bclaf3 and Prkra as regulators of Wnt-independent gastric epithelial differentiation^91^. Using an iPS cell-based pancreatic lineage differentiation model, Huangfu and colleagues demonstrated that HHEX serves as a gatekeeper for pancreatic lineage specification, emphasizing its pivotal role in organ development^92^.

## Outlook

### Beyond simple viability screens: perturbomics in more complex systems

Most perturbomics studies published so far rely on oversimplified experimental models, such as two-dimensional cultured cancer cell lines. This limitation stems from the scalability challenges of more complex systems, such as organoid cultures, which preclude large-scale pooled screens, and the technical difficulties of in vivo delivery of gRNA libraries to many tissues. However, advances in organoid and assembloid culture systems, as well as in vivo gRNA delivery techniques, are now enabling perturbomics studies in more physiologically relevant contexts. These advancements hold particular promise for uncovering genes involved in developmental defects. For instance, Pasca and colleagues used human iPS cell-derived forebrain assembloids—comprising subpallial and cortical organoids—to identify SMAD4, CSDE1, TERF2 and LNPK as modulators of interneuron development and migration, with implications for autism spectrum disorder^93^.

Perturbomics studies are increasingly integrating lineage tracing techniques and single-cell multiomics to provide a comprehensive view of cellular phenotypes upon gene perturbation. For example, CRISPR screens combined with lineage tracing enable detailed analyses of lineage expansion at the single-cell level. Knoblich and colleagues used human ES cells with unique barcodes and gRNA libraries to generate cerebral organoids, analyzing clonal expansion of specific barcoded cells. They identified IER3IP1 as a key regulator of microcephaly^94^. Further work by the same group, combining lineage tracing with scRNA-seq in cerebral organoids, identified ARID1B as crucial for brain development and the radial glia-to-oligodendrocyte precursor cell transition, with implications for autism spectrum disorder^95^. Furthermore, cellular indexing of transcriptomes and epitopes sequencing (CITE-seq) has been adapted to perturbomics. A technique known as Perturb-CITE-seq simultaneously detects epitopes, RNA and gRNA in single cells. This approach was applied to patient-derived tumor–tumor-infiltrating lymphocyte cocultures, revealing that the loss of CD58 promotes IFN-γ-independent immune evasion^96^.

### Perturbomics in the era of AI

Recent advances in artificial intelligence (AI) are transforming biological research, enabling integrative data processing and uncovering hidden insights from large datasets. In cancer research, AI has demonstrated considerable potential for enhancing predictive accuracy and generating novel insights. For instance, the deep learning-based MSIntuit tool detects microsatellite instability, a key biomarker in colorectal cancer, from hematoxylin–eosin-stained slides of patients’ samples with a sensitivity of 0.96–0.98 (ref. ^97^). Similarly, machine learning models have shown promise in early pancreatic cancer detection using clinical records^98^, while biology-guided deep learning applied to preoperative computed tomography images has provided prognostic predictions for gastric cancer and potential immunotherapy responses^99^.

Data-rich perturbomics studies provide a robust foundation for developing AI-based models that predict phenotypic changes upon gene perturbation. Leskovec and colleagues, for instance, developed the graph-enhanced gene activation and repression simulator (GEARS), which integrates CRISPRa screening data with previously published Perturb-seq datasets to predict combinatorial perturbation effects on gene interactions^100^. Generative machine learning algorithms utilized Perturb-Seq data to identify key perturbational signatures in RNA sequencing data exposed to spaceflight conditions obtained from the NASA Open Science Repository^101^. In addition, a deep learning-based pooled optical screening method has elucidated relationships between cellular morphology and gene function^102^. Collectively, these innovations highlight the transformative role of AI in high-resolution, data-driven exploration of genetic, cellular and cancer-related phenomena.

A decade of perturbomics research leveraging CRISPR screens has yielded profound insights into the pathogenesis of diverse diseases and identified potential therapeutic target genes. Continued advancements in CRISPR-based technologies—particularly their integration with lineage tracing, single-cell transcriptomics and more physiologically relevant models—hold promise to further enhance our understanding of complex biological systems and their implications for human health.
